# A Feeder-Bus Dispatch Planning Model for Emergency Evacuation in Urban Rail Transit Corridors

**DOI:** 10.1371/journal.pone.0161644

**Published:** 2016-09-27

**Authors:** Yun Wang, Xuedong Yan, Yu Zhou, Wenyi Zhang

**Affiliations:** 1 MOE Key Laboratory for Urban Transportation Complex System Theory and Technology, School of Traffic and Transportation, Beijing Jiaotong University, Beijing, China; 2 State Key Lab of Rail Traffic Control and Safety, Beijing Jiaotong University, Beijing, China; Lanzhou University of Technology, CHINA

## Abstract

The mobility of modern metropolises strongly relies on urban rail transit (URT) systems, and such a heavy dependence causes that even minor service interruptions would make the URT systems unsustainable. This study aims at optimally dispatching the ground feeder-bus to coordinate with the urban rails’ operation for eliminating the effect of unexpected service interruptions in URT corridors. A feeder-bus dispatch planning model was proposed for the collaborative optimization of URT and feeder-bus cooperation under emergency situations and minimizing the total evacuation cost of the feeder-buses. To solve the model, a concept of dummy feeder-bus system is proposed to transform the non-linear model into traditional linear programming (ILP) model, i.e., traditional transportation problem. The case study of Line #2 of Nanjing URT in China was adopted to illustrate the model application and sensitivity analyses of the key variables. The modeling results show that as the evacuation time window increases, the total evacuation cost as well as the number of dispatched feeder-buses decrease, and the dispatched feeder-buses need operate for more times along the feeder-bus line. The number of dispatched feeder-buses does not show an obvious change with the increase of parking spot capacity and time window, indicating that simply increasing the parking spot capacity would cause huge waste for the emergent bus utilization. When the unbalanced evacuation demand exists between stations, the more feeder-buses are needed. The method of this study will contribute to improving transportation emergency management and resource allocation for URT systems.

## Introduction

To deal with the serious metropolitan problems of traffic congestion, the limitation in land use and environmental contamination, the speed and scale of urban rail transit (URT) development in China are far surpassing anywhere else in the world [1–3]. By the end of 2013, fifteen cities in China have built up URT systems with the total operation length of 2408 kilometers [4]. Characterized by a high level of safety, large capacity, wide accessibility, high speed and energy performance [5], URT has been utilized as a key solution for supporting mobility needs in high-density urban areas, and the total passenger volume of URT has even reached up to nearly eleven billion trips in China [4].

Because the public transportation in high-density urban areas heavily depends on URT systems, even minor operation disruptions in URT system can result in serious economic losses and social chaos [6]. The operation disruptions might be attributed to divers unexpected events, e.g., infrastructure malfunctions, trampling accidents, fire emergency and extreme weather conditions, etc. [7–8]. For example, in China, a rear-end collision occurred in Shanghai Metro Line #10 on September 27^th^, 2011. The emergency happened at 2:10 pm and the stranded passengers were not totally evacuated until 8:38 pm, indicating that the influence of the emergency lasted for more than 6 hours. Another rear-end collision occurred in Shanghai Metro Line #1 on December 22^th^, 2009, which caused more than 4 hours’ service disruptions.

Proper and flexible emergency countermeasures can largely reduce the side effects of emergency, such as potential operational dangers, passengers’ delay and complaints, and so on. URT and bus transportation, as the two major layers of the urban public transportation system, can be collaboratively operated for emergency response in URT corridors [9]. Without timely dispatching feeder-buses to evacuate passengers gathering outside URT stations, the local urban ground transportation operation would be substantially influenced and secondary incident risk would potentially increase. Through introducing localized integration with bus services, feeder-buses cooperating with URT can indeed realize the connection of transportation under emergent conditions [10]. Moreover, it can effectively enhance the resilience of URT to disruptions and simultaneously promote the serviceability of public transportation system [8, 10–12]. This paper addresses the issues in the feeder-bus route design and resource allocation for emergency evacuation in urban rail transit corridors, which is one of the most significant aspects in the contingency plan for urban public transportation management. The contribution of this study lies in the following aspects:

- A trains-buses-trains cooperation mode is put forward to eliminate the effect of unexpected service interruptions in URT corridors. In fact, when an emergent event occurs at a point along a certain URT line and breaks its operation, the URT line can be divided into two sub-lines which can still keep running independently by utilizing turn-back line. Thus, through integrating the local bus services, feeder-buses can be dispatched to the influenced stations and served as a ferry system to connect the two partial routings of URT line. Further, a trains-buses-trains operation mode can temporally maintain the URT function during emergency. In other words, feeder-buses cooperating with URT can indeed realize the connection of transportation and ensure passengers’ smooth transfer between the systems.
- A mathematical feeder-bus dispatch planning model is established to optimize the feeder-bus dispatch scheme for emergency evacuation in URT corridors. In the co-operation system, the collaborative adjustments in both URT system and feeder-bus dispatch scheme are required for URT and bus integration. The model aims at designing an optimal and operational scheme for sets of parking spots, feeder-buses, and URT stations. The objective of this study is to minimize the total travelling time of feeder-buses within a required evacuation time period. Thus, the decision makers can determine the number of feeder-buses that need to be dispatched, which bus parking spots the feeder-buses should be dispatched from, which URT stations the feeder-buses should be dispatched to, and the operation times of feeder-buses running between demand stations.
- A concept of dummy feeder-bus system is proposed to capture the optimal solution of the feeder-bus dispatch planning model. The original proposed planning model is non-linear integer programming (NLIP) which is hard to obtain the optimal solution. Thus, in the process of model solution, a concept of dummy feeder-bus system is proposed to translate this NLIP problem into a traditional linear integer programming (LIP), i.e., transportation problem. The proposed model solution approach can be carried out in a computationally efficient way in order to provide a quick response plan.
- A case study based on the Nanjing public transit system was carried out to demonstrate the practical significance of the proposed method. The results show that such a method is feasible to utilize the ground bus services to evacuate those influenced passengers during URT operation disruptions.

The remainder of this study is organized as follows. Section 2 reviews relevant studies in the literature. Section 3 develops a modeling framework for planning the feeder-bus dispatch for emergency evacuation in URT corridors, including problem statement and optimization model construction. Section 4 introduces the solution method of the model. In section 5, a case study based on the Line #2 of Nanjing URT system is performed to illustrate the model application. Finally, Section 6 provides conclusions and suggests future research directions.

## Literature Review

The URT and feeder-bus service integration problem (UFSIP) has been a research hotspot for several decades. Sivakumaran et al. [13] pointed out that introducing integrated operation between URT and bus in a multi-level public transit system could bring great benefits for both transit operators and users. A good integrated transit system can efficiently eliminate the duplicated routes, expand the service coverage, and improve the service quality and passengers’ satisfaction level [14–15].

Previous studies about UFSIP generally pay more attention to the optimization of feeder-bus operation, since the structure of URT system under normal traffic conditions is usually set as a given scenario [16]. Literature on feeder-bus optimization problem ranges from strategic planning to operational management. In the planning stage, the feeder-bus network structure and feeder-bus routes should be designed based on some specified objectives. Particularly, Kuah and Perl [17] first defined the feeder-bus network design problem (FNDP), and presented a mathematical programming model to obtain a reasonable feeder-bus network to access a given existing URT system. Current et al. [18] and Kuan et al. [14] proposed that FNDP was a hierarchical problem while the URT routes were primary paths and the feeder-bus routes were secondary paths. Chien and Schonfeld [19] developed a joint optimization model for a new transit system with no existing bus routes but only one existing URT line, based on the assumption that bus routes were parallel with URT lines. Some other relevant papers include Ceder and Wilson [20], Li and Quadrifoglio [21], Chou et al. [22], Ciaffi et al. [23], Deng et al. [24], Vuchic [25]. Lin and Wong [26] firstly developed a multi-objective model to solve the feeder-bus route design problem (FRDP), which can obtain a compromise solution by comprehensively considering various stakeholder concerns, including service providers, bus users, non-bus road users. Almasi et al. [27] built up a transit services optimization model with feeder-bus and fixed rail lines, aiming to design a set of feeder-bus routes and determine operating frequency on each route. What makes their study outstanding is that instead of presenting with a single parameter, the cost for each route was divided into user cost, operation cost and social cost, and each of them were presented in a more detailed way. The heuristic algorithm became to be applied by Shrivastava and Dhingra [28] to optimize the feeder routes for buses to suburban railway stations. Readers may refer to Shrivastava and O’Mahony [29–31], Pan et al. [32], and Song and Liu [33] for comprehensive reviews. In the operation stage, optimally coordinated feeder-bus operation schedules and vehicle allocations should be determined according to the feeder-bus network and line routes. Salzborn [34] developed a mathematical model to calculate the service frequency of feeder-buses and Lee and Schonfeld [35] improved this model by taking the delay time into consideration. Li et al. [36] optimized the URT system with feeder-bus services for different optimization objectives, including social welfare maximization and profit maximization. Some other representative studies include Ceder and Golany [37], Chung [38], and Jiang and Huang [39].

Although a comprehensive body of literature on UFSIP is available, there still exist limitations and gaps in previous studies. Firstly, as previously mentioned, emergencies frequently occur in URT corridors due to a variety of reasons, and may cause serious consequences [40]. However, most scenarios of existing research on UFSIP are set under normal conditions, while few studies are available for emergent situations. Secondly, the approach proposed for normal conditions cannot be applied directly in this paper but the research emphases of UFSIP under normal and emergent conditions are quite different. The former mainly focuses on how to utilize feeder-buses to extend the service coverage of URT and improve the service quality of transit system in a long term. For the latter, when emergency occurs in URT corridors, the major task of feeder-buses is to evacuate stranded passengers and ensure transportation continuation within a certain required time period. Thus, the topic of UFSIP under emergent conditions has been receiving increasing attention. Representative studies include Kepaptsoglou and Karlaftis [41], Teng and Xu [10], Darmanin et al. [42], Jin et al. [43], Jin et al. [8], and Lv et al. [44]. Kepaptsoglou and Karlaftis [41] defined the problem as bus bridging service (i.e., feeder-bus service) and proposed a modeling framework to design the bus routes and resource allocation. Teng and Xu [10] designed a transportation capacity calculation method and optimize the site selection problem of feeder-bus. Darmanin et al. [42] proposed the disruption response strategies for utilizing feeder-bus services to transfer passengers to other rail lines, and a specific case of the Melbourne metro system was also conducted. Jin et al. [8] developed a methodological framework with two hierarchical steps for planning and designing an efficient feeder-bus network: designing of feeder-bus routes and feeder-bus resource allocation among the routes. Lv et al. [44] developed an evacuation planning model in response to bus–subway corridor incidents based on the interval chance-constrained integer programming (EICI) method. They proposed a recovery approach to complement the URT service disruptions during which the temporary feeder-buses should be dispatched to the affected areas in URT system. In this study, we have also used the similar recovery strategies, but with a more integrated approach and an entirely different method. Whereas Lv et al. provided two choices for passengers to continue their travel, including shuttle buses and routine buses, we propose dispatching feeder-buses to the influenced URT stations which can serve as a ferry system to connect the two partial routings of URT line. Therefore, passengers can have a smooth transfer without spending the walk time to the original bus stations. Additionally, no previous studies have paid attention to the influence of the feeder-bus parking spot locations on the design of feeder-bus network, route choice and service frequency. Actually, the distance between feeder-bus parking spots and URT stations profoundly affects the efficiency of feeder-bus dispatch and evacuation cost. Therefore, in this study, the available number of feeder-buses and their parking spot distribution were set as key parameters.

Based on the above literature analyses, many researchers have made an attempt to design a more efficient integration system between feeder-bus and URT system. Table 1 provides a systematic comparison of key model components and solution methods on the URT and feeder-bus service integration problem in the existing literature.

**Table 1:** Comparisons of Key Model Components and Solution Methods on the URT and Feeder-Bus Service Integration Problem in the Existing Literature.

Applicable scenarios	Modeling characteristics	Objective Function	Solution Method	Results	Publication
NC, SS	IP, MOP,	Multiple objectives: maximize service coverage for designed feeder-bus routes; minimize the maximum route travel time of all routes; minimize the total length of planned feeder-bus routes	TOPSIS approach	RD	Lin and Wong (2014)
NC, SL	IP	Minimize the total costs of operators, users and society	Metaheuristic Algorithm (GA, PSO, ICA)	RD, SF	Mohammad et al (2014)
NC, ML	AM, SOP	Minimize the total costs of users and operators	Hybrid approach using GA and *k-*path algorithm	RD, TD	Prabhat et al (2009)
NC, ML	AM	Minimize path length	Heuristic algorithm	RD	Prabhat et al (2001)
NC, SS	IP, MOP	Minimize the total costs of users and operators	Gravity-based method	RD	Pan et al (2014)
NC	AM	Combination of measures of performance	CPLEX	PS	Li and Luca (2009)
EC, ML	MIP	Minimize the increase in passengers’ travel time	Column Generation solved by CPLEX	RD, VA	Jin et al (2015)
EC, ML	MIP	Minimize passengers’ travel time	Two stage stochastic program	RD, VA	Jin et al (2014)
EC, SL	MIP	Minimize total evacuation time	Transfer to two submodels	RD, VA	Lv et al (2015)
NC	IP	Minimize total cost	Heuristic algorithm	ND	Kuah et al (1989)
NC, ML	MIP	Minimize the total costs of suppliers and users	Exhaustive Search Algorithm	RD	Chien and Yang (2000)
NC, ML	MOP	Minimize the route length and maximize the bus frequency	Heuristic route generation algorithm and GA	ND	Ciaffi et al (2012)
EC, SL, MS	NIP	Minimum total evacuation cost	Transform the NIP model into IP model with the concept of dummy feeder-bus parking spot, and solved by Lingo	RD, VA, SF	Our Paper

**Modeling scenarios:** NC- Normal Conditions; EC-Emergent Conditions; SS-Single Station; SL- Single Line; MS- Multiple Stations; ML- Multiple Line; **Modeling characteristics:**IP-Integer Programming; NIP—Non-Integer Programming; MIP- Mixed Integer Programming; SOP- Single Objective Problem; MOP- Multi-Objective Problem; NM-Network Mode; **Results:** ND–Network Design; RD–Route Design; VA–Vehicle Allocation; TD–Timetable Design; SF–Service Frequency;

## Modeling Framework

### Nomenclature

Some symbolic notations used in the feeder-bus dispatch model are defined in Table 2.

**Table 2:** Notations Used in the Paper.

***Sets*:**		
*S*	set of URT stations, *s* ∈ *S*, *S* = {*s* | *s* = *s*_1_, *s*_2_, …, *s*_*l*_, …, *s*_*m*_, …, *s*_*n*_}, where *n* is the total number of URT stations	
*ES*	set of endpoint stations, *es* ∈ *ES*, *ES* = {*es | es* = es_*1*_, *es*_2_}, where es_*1*_ = s_*1*_ = 1 and *es*_2_ = *s*_*n*_ = *n*	
*DS*	set of demand stations, *ds* ∈ *DS*, *DS* = {*ds | ds* = ds_*1*_, *ds*_2_}, where *ds*_1_ = *s*_*l*_ = *l* and *ds*_2_ = *s*_*m*_ = *m*	
*MS*	set of middle stations, *ms* ∈ *MS*, *MS* = {*ms* | *ms* = *ms*_1_, *ms*_2_, …, *ms*_*c*_}, where *ms*_1_ = *s*_*l*+1_ = *l*+1 and *ms*_*c*_ = *s*_*m*−1_ = *m* − 1	
*FS*	set of feeder-bus stations, *fs* ∈ *FS*, *FS* = {*fs* | *fs* = *fs*_1_, *fs*_2_, …, *fs*_*b*_}, where *b = m − l* + 1	
*PS*	set of feeder-bus parking spots, *ps* ∈ *PS*, *PS* = {*ps* | *ps* = *ps*_1_, *ps*_2_, …, *ps*_*a*_}, where *a* is the total number of feeder-bus parking spots	
*D*_*j*_	sets of dummy feeder-bus parking spots, $D_{j}=\{d_{j}|d_{j}=d_{j\;}^{1},ps_{j}^{2},\ldots ,ps_{j}^{K_{1}^{j}+K_{2}^{j}+K_{3}^{j}+K_{4}^{j}+4}\}\;1\leq j\leq a$, where $K_{r}^{j}$ is the maximum operation time of feeder-buses dispatched from subsistent feeder-bus parking spot, *j*, where *j* = *ps*_*j*_ ∈ *PS*; $K_{1}^{j}+K_{2}^{j}+K_{3}^{j}+K_{4}^{j}+4$ is the total number of dummy feeder-bus parking spots of their subsistent feeder-bus parking spot *j*	
$R_{i}^{ps}$	set of the alternative operation routes of the feeder-bus, *i*, dispatched from parking spot, *ps*, $R_{i}^{ps}=\{r_{i}^{ps}|r_{i}^{ps}=r_{i1}^{ps},r_{i2}^{ps},r_{i3}^{ps},r_{i4}^{ps}\}$	
***Parameters*:**		***Unit***
*C*_*u*_	capacity of URT train	number of persons per train
*C*_*b*_	design seating capacity of feeder-bus	number of persons per feeder-bus
$C_{b}^{*}$	design seating capacity of dummy feeder-bus	number of persons per feeder-bus
*C*^*ps*^	capacity of feeder-bus parking spot *ps*	number of vehicles per spot
*φ*_*u*_	load factor of URT trains	NA
*φ*_*b*_	load factor of feeder-buses	NA
*Q*_*s*_	number of passengers stranded in URT station *s* when the emergent event occurs	number of persons
*α_s_*_,*s*′_	ratio of passengers destining from station *s* to station *s*′	NA
*q*	number of passengers arriving at feeder-bus station *fs* from outside per hour	number of persons
*t*	time window after the emergency happens	hour
*h*	train departure interval of the partial routings	minute
*q*_*fs*_	section passenger volume in feeder-bus station *fs*	number of persons
*q*_max_	maximum section passenger volume	number of persons
$t_{fs}^{ps}$	minimum journey time from parking spot *ps* to feeder-bus station *fs*	minute
*t*_*fs*_	journey time between feeder-bus stations *fs*_1_ and *fs*_*b*_	minute
$t_{i}^{ps}$	total travelling time of feeder-bus, *i*, dispatched from parking spot *ps*	minute
$K_{r}^{ps}$	maximum operation times between two terminal feeder-bus stations of the feeder-buses dispatched from parking spot *ps* and operate along Route *r*	number of times
ℕ	nonnegative integers	NA
***Decision variables*:**		***Unit***
$x_{i}^{ps}$	a bivariate variable identifying whether the feeder-bus, *i*, in parking spot, *ps*, is dispatched or not	NA
$x_{j}^{i}$	number of dispatched dummy feeder-buses from the No. *i* dummy feeder-bus parking spot of the subsistent feeder-bus parking spot, *j*	number of vehicles
$r_{i}^{ps}$	operation route of feeder-bus, *i*, dispatched from parking spot *ps*	NA
$k_{i}^{ps}$	operation times between two terminal feeder-bus stations of the feeder-bus, *i*, dispatched from parking spot *ps*	number of times

### Problem statement

**Fig 1:**
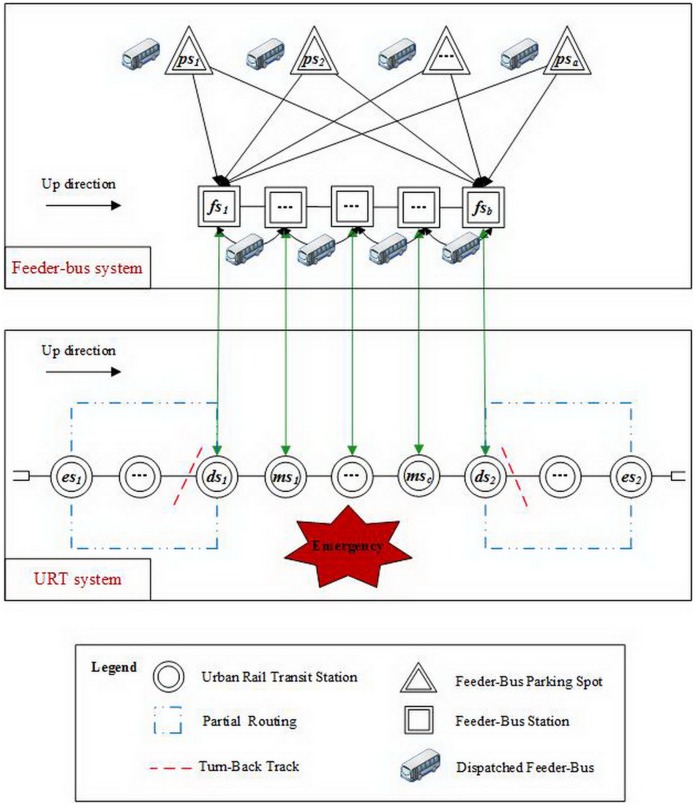
Schematic diagram of feeder-bus co-scheduling under URT emergency.

Fig 1 illustrates the process of feeder-bus dispatch for emergency evacuation in URT corridors. In the URT corridor, let *ES* = {*es* | *es* = *es*_1_, *es*_2_} denote a finite set of endpoint stations. Station *es*_1_ and station *es*_2_ are respectively the first and the last stations in the up direction while station *es*_2_ and station *es*_1_ are respectively the first and the last stations in the down direction. Let *DS* = {*ds* | *ds* = *ds*_1_, *ds*_2_} represent a finite set of demand stations which are equipped with the turn-back track. Below *MS* = {*ms* | *ms* = *ms*_1_, *ms*_2_, …, *ms*_*c*_} represents a finite set of middle stations (stations influenced by emergency), and the emergency occurs in the middle station *ms*_*m*_. In the feeder-bus system, let *PS* = {*ps* | *ps* = *ps*_1_, *ps*_2_, …, *ps*_*a*_} represent a finite set of feeder-bus parking spots. Below *FS* = {*fs* | *fs* = *fs*_1_, *fs*_2_, …, *fs*_*b*_} represents a finite set of feeder-bus stations where feeder-buses would be dispatched to. There is a one-to-one correspondence relationship between feeder-bus stations and demand stations. In particular, the feeder-bus stations *fs*_1_ and *fs*_*b*_ are located at the demand stations *ds*_1_ and *ds*_2_ respectively.

When an emergent event occurs in middle station *ms*_*m*_, it breaks the normal operation of URT. The feeder-bus dispatch scheme should be generated for ferrying the stranded passengers between the two URT sub-lines: one is between the endpoint station *es*_1_ and the demand station *ds*_1_, and the other is between the endpoint station *es*_2_ and the demand station *ds*_2_. By utilizing the turn-back line equipped in the demand stations *ds*_1_ *and ds*_2_, the two sub-lines can still keep running independently. Furthermore, feeder-buses will be dispatched to feeder-bus stations *fs*_1_ and *fs*_*b*_, i.e., the demand stations *ds*_1_ and *ds*_2_, and operate circularly between the two feeder-bus stations. As a ferry system, the feeder-buses can carry passengers to arrive at their destination directly or transfer to the two partial routings of URT.

During the cooperation process, the feeder-buses should first depart from the parking spot, *ps*, and run to the feeder-bus stations *fs*_1_ and *fs*_*b*_. Then, the feeder-buses go through a series of feeder-bus stations between *fs*_1_ and *fs*_*b*_, and operate several times between the feeder-bus stations *fs*_1_ and *fs*_*b*_. Finally, the feeder-buses return back to their original parking spot, *ps*, from either feeder-bus station *fs*_1_ or *fs*_*b*_. Moreover, passengers can get on and off the feeder-buses at each feeder-bus station *fs*.

**Fig 2:**
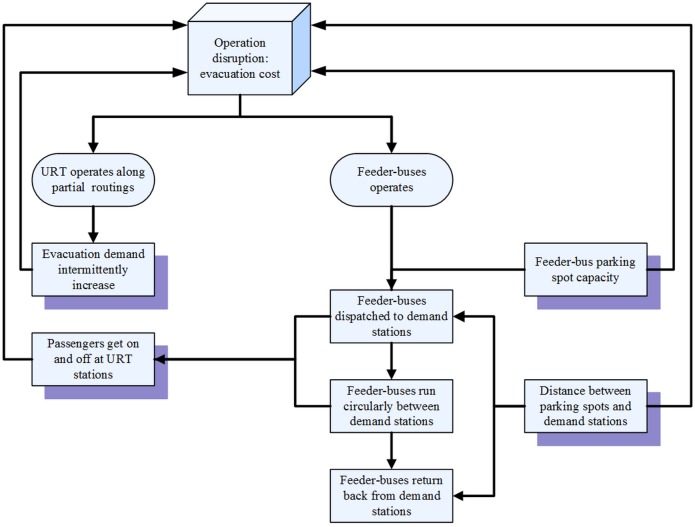
Schematic diagram for feeder-bus emergency evacuation in URT corridors.

As shown in Fig 2, the design of feeder-bus dispatch scheme is a systematic problem, which is determined by parameters related to both the URT system and feeder-bus system, such as evacuation demands, capacity of feeder-bus parking spots and feeder-buses’ travelling time. Due to the operation of URT partial routings, the passenger volume in demand stations will intermittently increase. The scheme of feeder-bus dispatch should be formulated based on the real evacuation demands and the capacity of feeder-bus parking spots.

### Basic assumption

Several assumptions made throughout the paper for simplicity in the model formulation are explained as follows:

#### Assumption 1

*α*_*s*,*s*′_, the ratio of passengers destining from station *s* to station *s*′, can be obtained based on the historical OD distribution data, where $\sum_{s\prime \in s}\alpha_{s,s\prime }=1$.

#### Assumption 2

The feeder-bus journey time, *t*_*fs*_, between feeder-bus stations *fs*_1_ and *fs*_*b*_ is deterministic according to the bus running speed, road conditions, the number of passengers getting on and off in stations, etc. in the local road networks. With fixed feeder-bus running times, the proposed model can be simplified significantly. Similarly, the journey time, $t_{fs}^{ps}$, between feeder-bus parking spot, *ps*, and feeder-bus station, *fs*, is also deterministic.

#### Assumption 3

All the stranded passengers are evacuated by the feeder-buses. Note that the private cars and taxis are not considered in this study.

### Model construction

#### Maximum section passenger volume along the feeder-bus line

Maximum section passenger volume along the feeder-bus line is a key parameter for calculating the demand number of feeder-buses, which is equal to the maximum of the section passenger volume among all feeder-bus stations.

**Fig 3:**
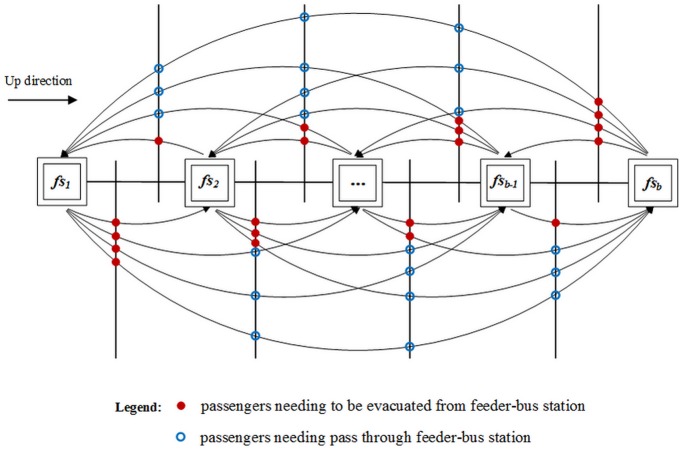
Schematic diagram of feeder-bus co-dispatching scheme.

As is shown in Fig 3, the section passenger volume, *q*_*fs*_, in feeder-bus station, *fs*, may contain two categories: passengers who need to be evacuated from the feeder-bus station *fs*, ${\bar{q}}_{fs}$, and passengers who need to pass through the feeder-bus station *fs*, ${\hat{q}}_{fs}$. Further, passengers who need to be evacuated from the feeder-bus station *fs*, ${\bar{q}}_{fs}$, contains both the original stranded passengers, ${\bar{q}}_{fs,\;O}$, and subsequent arriving passengers, ${\bar{q}}_{fs,\;A}$.

In the up direction, Eq (1) describes the section passenger volume, $q_{fs_{1}}^{1}$, of feeder-bus station *fs*_1_. Note that feeder-bus station *fs*_1_ is the departure station of feeder-bus line. So there is no passenger needing pass through feeder-bus station *fs*_1_. Therefore, the section passenger volume, $q_{fs_{1}}^{1}$, of feeder-bus station *fs*_1_ is equal to the number of passengers who need to be evacuated from the feeder-bus station *fs*_1_, ${\bar{q}}_{fs_{1}}^{1}$, which intermittently increases due to the operation of partial routings. Moreover, the time interval of the stranded passengers’ intermittently increasing is determined by the departure interval of URT. In particular, as is shown in Eq (2), the original stranded passengers, ${\bar{q}}_{fs_{1},\;O}^{1}$, contains the passengers from feeder-bus station *fs*_1_ to the downstream stations. The subsequent arriving passengers ${\bar{q}}_{fs_{1},\;A}^{1}$ can be obtained by Eq (3), where [*t* / *h*^1^] is the number of train arriving times within the time window.

$$q_{fs_{1}}^{1}={\bar{q}}_{fs_{1}}^{1}={\bar{q}}_{fs_{1},\;O}^{1}+{\bar{q}}_{fs_{1},\;A}^{1}$$ (1)

$${\bar{q}}_{fs_{1},\;O}^{1}=Q_{fs_{1}}*\sum_{s>fs_{1},\;s\in S}\alpha_{fs_{1},\;s}$$ (2)

$$\begin{array}{ll} {\bar{q}}_{fs_{1},\;A}^{1} & =\underset{s<fs_{1},s\in S}{\sum }(Q_{s}*\underset{s\prime >fs_{1},s,s\prime \in S}{\sum }\alpha_{s,s\prime })\\ & \;+(C_{u}^{*}*\varphi_{u}*\left[t/h^{1}\right]+q*t)*\underset{s<fs_{1},s\prime >fs_{1},s,s\prime \in S}{\sum }\alpha_{s,s\prime } \end{array}$$ (3)

In the up direction, except for station *fs*_*b*_, feeder-bus station *fs*_2_ and its downstream stations contain both passengers needing to be evacuated from the feeder-bus station and passengers needing pass through. Eq (4) describes the section passenger volume, $q_{fs_{i}}^{1}$, of feeder-bus station *fs*_*i*_, where l < *i* < *b*. Eq (5) describes the number of passengers needing to be evacuated from the feeder-bus station *fs*_*i*_, while the original stranded passengers, ${\bar{q}}_{fs_{i},\;O}^{1}$, contain the passengers from feeder-bus station *fs*_*i*_ to the downstream stations and ${\bar{q}}_{fs_{i},\;A}^{1}$ contain the subsequent arriving passengers with destinations of downstream stations. As shown in Eq (6), the number of passengers needing pass through feeder-bus station *fs*_*i*_, ${\hat{q}}_{fs_{i}}^{1}$, is equal to the sum of passengers from station *fs* to station *fs*′ while station *fs* is before station *fs*_*i*_ and station *s*′ is behind station *fs*_*i*_.

$$q_{fs_{i}}^{1}={\bar{q}}_{fs_{i}}^{1}+{\hat{q}}_{fs_{i}}^{1}$$ (4)

$${\bar{q}}_{fs_{i}}^{1}={\bar{q}}_{fs_{i},\;O}^{1}+{\bar{q}}_{fs_{i},\;A}^{1}=(Q_{fs_{i}}+q*t)*\sum_{s>fs_{i},\;s\in S}\alpha_{fs_{i},s}$$ (5)

$${\hat{q}}_{fs_{i}}^{1}=q_{fs_{1}}^{1}*\sum_{s\prime >fs_{1},s\prime \in S}\alpha_{fs_{1},s\prime }$$ (6)

Therefore, the maximum section passenger volume in the up direction can be described as Eq (7).

$$q_{\text{max}}^{1}=\text{max}\left\{q_{fs_{1}}^{1},q_{fs_{2}}^{1},\ldots ,q_{fs_{b-1}}^{1}\right\}.$$ (7)

Similarly, in the down direction, Eqs (8) and (9) respectively describe the section passenger volume in feeder-bus station, *fs*_*b*_ and its downstream feeder-bus station *fs*_*i*_, where l < *i* < *b*. The maximum section passenger volume in the down direction can be described as Eq (10).

$$\begin{array}{ll} q_{fs_{b}}^{2} & ={\bar{q}}_{fs_{b}}^{2}={\bar{q}}_{fs_{b},\;O}^{2}+{\bar{q}}_{fs_{b},\;A}^{2}\\ & =Q_{fs_{b}}*\sum_{s<fs_{b},\;s\in S}\alpha_{fs_{b},\;s}+\sum_{s>fs_{b},s\in S}(Q_{s}*\sum_{s\prime <fs_{b},s,s\prime \in S}\alpha_{s,s\prime })\\ & \;+(C_{u}^{*}*\varphi_{u}*\left[t/h^{2}\right]+q*t)*\sum_{s>fs_{b},s\prime <fs_{b},s,s\prime \in S}\alpha_{s,s\prime } \end{array}$$ (8)

$$\begin{array}{ll} q_{fs_{i}}^{2} & ={\bar{q}}_{fs_{i}}^{2}+{\hat{q}}_{fs_{i}}^{2}={\bar{q}}_{fs_{i},\;O}^{2}+{\bar{q}}_{fs_{i},\;A}^{2}+{\hat{q}}_{fs_{i}}^{2}\\ & =(Q_{fs_{i}}+q*t)*\sum_{s<fs_{i},\;s\in S}\alpha_{fs_{i},s}+q_{fs_{b}}^{2}*\sum_{s\prime <fs_{b},s\prime \in S}\alpha_{fs_{b},s\prime } \end{array}$$ (9)

$$q_{\text{max}}^{2}=\text{max}\left\{q_{fs_{2}}^{2},q_{fs_{3}}^{2},\ldots ,q_{fs_{b}}^{2}\right\}$$ (10)

#### Constraints analyses

(1) Alternative feeder-bus operation routes

**Fig 4:**
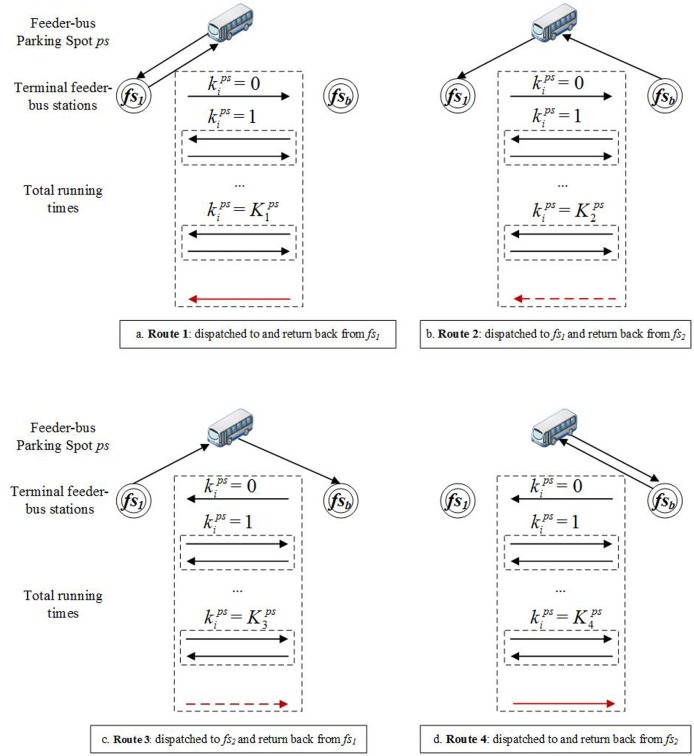
Four alternative operation routes of feeder-buses.

The feeder-buses dispatched to the feeder-bus stations *fs*_1_ and *fs*_*b*_ can return back to their original parking spot from either station *fs*_1_ and *fs*_*b*_. Therefore, as shown in Fig 4, all the feeder-buses have four alternative operation routes. Particularly, Route 1 means the feeder-buses are both dispatched to and return back from the feeder-bus station *fs*_1_; Route 2 means the feeder-buses are dispatched to the feeder-bus station *fs*_1_ and return from *fs*_*b*_; Route 3 means the feeder-buses are dispatched to the feeder-bus station *fs*_*b*_ and return from *fs*_1_; and Route 4 means the feeder-buses are both dispatched to and return back from the feeder-bus station *fs*_*b*_. Eq (11) describes that $r_{i}^{ps}$ is a binary variable. When the feeder-bus *i* in the parking spot *ps* is dispatched, the value of $r_{i}^{ps}$ is one or otherwise zero. It also restrains that each dispatched feeder-bus can choose only one operation route.

$$r_{i}^{ps}=\sum_{r_{ij}^{ps}\in R_{i}^{ps}}r_{ij}^{ps}=\left\{ \begin{array}{l} 0\ldots \ldots feeder-bus\;i\;in\;parking\;spot\;ps\;is\;not\;dispatched\\ 1\ldots \ldots feeder-bus\;i\;in\;parking\;spot\;ps\;is\;dispatched \end{array}\right.$$ (11)

(2) Feeder-bus parking spot capacity limitation

Eq (12) describes that the total number of feeder-buses dispatched from parking spot *ps* ∈ *PS* should be less than the useable feeder-buses, *C*^*ps*^, in the parking spot *ps*. In particular, $\sum_{1\leq i\leq C^{ps}}x_{i}^{ps}$ adds up the total number of feeder-buses dispatched to the feeder-bus stations *fs*_1_ and *fs*_*b*_.

$$\sum_{1\leq i\leq C^{ps}}x_{i}^{ps}\leq C^{ps}\;(ps=1,\;2,\;\ldots \;,\;a)$$ (12)

(3) Evacuation capacity limitation of dispatched feeder-bus

As noted, the dispatched feeder-buses can operate for several times, $k_{i}^{ps}$, along the feeder-bus line. Each dispatched feeder-bus operating along Route 1 and Route 4 can respectively evacuate $(1+k_{i}^{ps})*C_{b}*\varphi_{b}$ passengers for both the up direction and the down direction. Each dispatched feeder-bus operating along Route 2 can evacuate $(1+k_{i}^{ps})*C_{b}*\varphi_{b}$ for the up direction and $k_{i}^{ps}*C_{b}*\varphi_{b}$ for the down direction. Each dispatched feeder-bus operating along Route 3 can evacuate $k_{i}^{ps}*C_{b}*\varphi_{b}$ passengers for the up direction and $(1+k_{i}^{ps})*C_{b}*\varphi_{b}$ for the down direction. Below Table 3 list the detail supporting capacity for each direction of each feeder-bus along different operation routes.

**Table 3:** The Supporting Capacity of Each Dispatched Feeder-Bus.

Operation route	Up direction	Down direction
$r_{i1}^{ps}=1$	$(1+k_{i}^{ps})*C_{b}*\varphi_{b}$	$(1+k_{i}^{ps})*C_{b}*\varphi_{b}$
$r_{i2}^{ps}=1$	$(1+k_{i}^{ps})*C_{b}*\varphi_{b}$	$k_{i}^{ps}*C_{b}*\varphi_{b}$
$r_{i3}^{ps}=1$	$k_{i}^{ps}*C_{b}*\varphi_{b}$	$(1+k_{i}^{ps})*C_{b}*\varphi_{b}$
$r_{i4}^{ps}=1$	$(1+k_{i}^{ps})*C_{b}*\varphi_{b}$	$(1+k_{i}^{ps})*C_{b}*\varphi_{b}$

Therefore, the total capacity for the up direction is $\sum_{ps\in PS}\sum_{1\leq i\leq C^{ps}}[(1+k_{i}^{ps})(r_{i1}^{ps}+r_{i2}^{ps}+r_{i4}^{ps})+k_{i}^{ps}r_{i3}^{ps}]*C_{b}*\varphi_{b}$, and that for the down direction is $\sum_{ps\in PS}\sum_{1\leq i\leq C^{ps}}[(1+k_{i}^{ps})(r_{i1}^{ps}+r_{i3}^{ps}+r_{i4}^{ps})+k_{i}^{ps}r_{i2}^{ps}]*C_{b}*\varphi_{b}$.

Eqs (13) and (14) restrain that the dispatched feeder-bus capacity of each direction should satisfy its feeder-bus demand.

$$\sum_{ps\in PS}\sum_{1\leq i\leq C^{ps}}[(1+k_{i}^{ps})(r_{i1}^{ps}+r_{i2}^{ps}+r_{i4}^{ps})+k_{i}^{ps}r_{i3}^{ps}]*C_{b}*\varphi_{b}\geq q_{\text{max}}^{1}$$ (13)

$$\sum_{ps\in PS}\sum_{1\leq i\leq C^{ps}}[(1+k_{i}^{ps})(r_{i1}^{ps}+r_{i3}^{ps}+r_{i4}^{ps})+k_{i}^{ps}r_{i2}^{ps}]*C_{b}*\varphi_{b}\geq q_{\text{max}}^{2}$$ (14)

(4) Limitation of feeder-bus operation times

As noted in assumption 2, the minimal journal time between the feeder-bus parking spots and feeder-bus stations, as well as that between two terminal feeder-bus stations are fixed. Therefore, within the time window, the limitations of the operation times between two terminal feeder-bus stations are determined by the operation routes of dispatched feeder-buses. Eq (15) respectively describes the theoretically maximum operation times of the above four operation routes, which is collectively determined by the time window, *t*, the journey time between feeder-bus parking spots and feeder-bus stations, $t_{fs}^{ps}$, and the time between the two terminal feeder-bus stations, *t*_*fs*_. Eq (16) restrains that the operation times of feeder-buses must be smaller than its theoretically maximum operation times.

$$\begin{array}{l} K_{1}^{ps}=\left[\frac{t-2*t_{fs_{1}}^{ps}-2*t_{fs}}{2\text{*}t_{fs}}\right]\ldots \ldots \ldots \ldots \ldots \ldots \ldots \ldots .r_{i1}^{ps}=1\\ K_{2}^{ps}=\left[\frac{t-t_{fs_{1}}^{ps}-t_{fs_{b}}^{ps}-t_{fs}}{2\text{*}t_{fs}}\right].\ldots \ldots \ldots \ldots \ldots \ldots \ldots \ldots .r_{i2}^{ps}=1\\ K_{2}^{ps}=\left[\frac{t-t_{fs_{b}}^{ps}-t_{fs_{1}}^{ps}-t_{fs}}{2\text{*}t_{fs}}\right].\ldots \ldots \ldots \ldots \ldots \ldots \ldots \ldots .r_{i3}^{ps}=1\\ K_{4}^{ps}=\left[\frac{t-2*t_{fs_{b}}^{ps}-2*t_{fs}}{2\text{*}t_{fs}}\right]\ldots \ldots \ldots \ldots \ldots \ldots \ldots \ldots r_{i4}^{ps}=1 \end{array}$$ (15)

$$k_{i}^{ps}\leq \left\{ \begin{array}{l} K_{1}^{ps}.\ldots \ldots \ldots \ldots \ldots \ldots \ldots \ldots .r_{i1}^{ps}=1\\ K_{2}^{ps}.\ldots \ldots \ldots \ldots \ldots \ldots \ldots \ldots .r_{i2}^{ps}=1\\ K_{2}^{ps}.\ldots \ldots \ldots \ldots \ldots \ldots \ldots \ldots .r_{i3}^{ps}=1\\ K_{4}^{ps}.\ldots \ldots \ldots \ldots \ldots \ldots \ldots \ldots .r_{i4}^{ps}=1 \end{array}\right.$$ (16)

Moreover, the number of operation times, $k_{i}^{ps}$, and its limitation, $K_{r}^{ps}$, should be nonnegative.

$$k_{i}^{ps}\in N$$ (17)

$$K_{r}^{ps}\in N$$ (18)

#### Optimization model

The feeder-bus dispatch planning model for emergency evacuation in an URT corridor can be formulated based on the analyses of above constraints, which aims to properly dispatch the feeder-buses for mitigating the influence of the emergency. The objective of the feeder-bus dispatch model is to minimize the total evacuation cost when targeting an evacuation time window. In this paper, the total evacuation cost is simplified as the total service time of all the dispatched feeder-buses.

As shown in Fig 4, the service time, $t_{i}^{ps}$, of each feeder-bus, *i*, in feeder-bus parking spot, *ps*, can be calculated by Eq (19).

$$t_{i}^{ps}=\left\{ \begin{array}{l} 2*t_{fs_{1}}^{ps}+(2+k_{i}^{ps})*t_{fs}\ldots \ldots \ldots \ldots \ldots \ldots \ldots \ldots r_{i1}^{ps}=1\\ t_{fs_{1}}^{ps}+(1+k_{i}^{ps})*t_{fs}+t_{fs_{b}}^{ps}\ldots \ldots \ldots \ldots \ldots \ldots \ldots r_{i2}^{ps}=1\;or\;r_{i3}^{ps}=1\\ 2*t_{fs_{b}}^{ps}+(2+k_{i}^{ps})*t_{fs}\ldots \ldots \ldots \ldots \ldots \ldots \ldots \ldots r_{i4}^{ps}=1 \end{array}\right.$$ (19)

Therefore, the service time of all the dispatched feeder-buses operating along Route 1 is $T_{1}=\sum_{ps\in PS}\sum_{1\leq i\leq C^{ps}}r_{i1}^{ps}*\left\{2*t_{fs_{1}}^{ps}+(2+k_{i}^{ps})*t_{fs}\right\}$, and that of Route 2, 3 and 4 are respectively $T_{2}=\sum_{ps\in PS}\sum_{1\leq i\leq C^{ps}}r_{i2}^{ps}*\left\{t_{fs_{1}}^{ps}+(1+k_{i}^{ps})*t_{fs}+t_{fs_{b}}^{ps}\right\}$, $T_{3}=\sum_{ps\in PS}\sum_{1\leq i\leq C^{ps}}r_{i3}^{ps}*\left\{t_{fs_{1}}^{ps}+(1+k_{i}^{ps})*t_{fs}+t_{fs_{b}}^{ps}\right\}$, and $T_{4}=\sum_{ps\in PS}\sum_{1\leq i\leq C^{ps}}r_{i4}^{ps}*\left\{2*t_{fs_{b}}^{ps}+(2+k_{i}^{ps})*t_{fs}\right\}$

The total service time of all the feeder-buses can be uniformly defined as *T*_1_+*T*_2_+*T*_3_+*T*_4_. Eq (20) aims to minimize the sum of all the feeder-buses’ total travelling time.

$$\text{min}T=T_{\text{1}}\text{+}T_{\text{2}}\text{+}T_{\text{3}}\text{+}T_{\text{4}}$$ (20)

## Model Solution

The optimization problem of feeder-bus dispatch under the URT corridor’s emergency can be transferred into a traditional transportation problem based on integer linear programming (ILP), which is typically characterized as follows:

1. A set of *supply points*: feeder-bus parking spots *PS*, from which feeder-buses are dispatched.
2. A set of *demand points*: feeder-bus stations *FS*, to which the feeder-buses are dispatched.
3. Each unit produced at a supply point *ps* and transported to a demand point *fs* incurs a variable cost of *c*.

**Fig 5:**
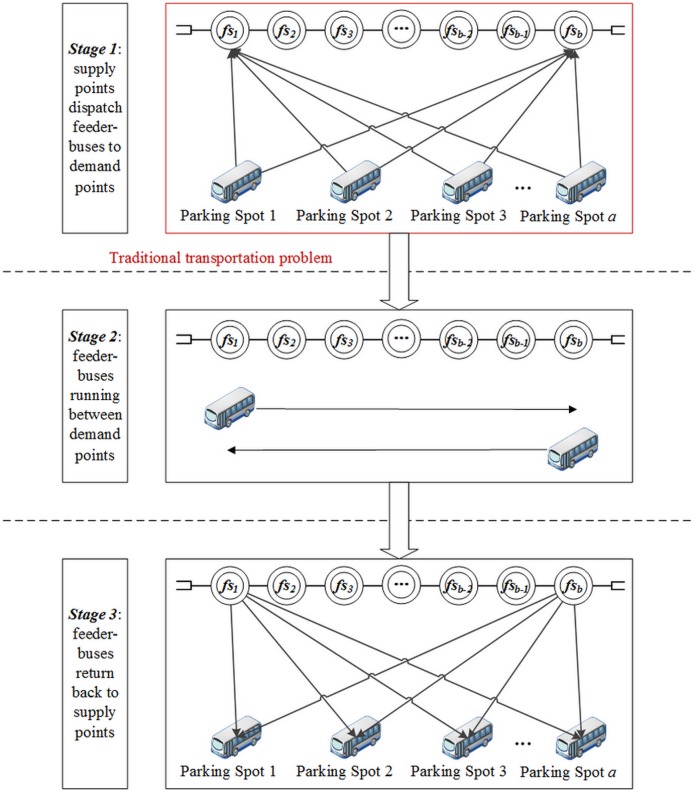
The processes of feeder-bus dispatch problem and transportation problem.

As is shown as Fig 5, the feeder-bus dispatch problem can be divided into three stages. In the first stage, the feeder-buses in the parking spots are dispatched from supply points to demand points; in the second stage, the feeder-buses are severed as ferry buses travelling cyclically between the two demand points to connect the disrupted URT systems; in the third stage, all the dispatched feeder-buses return back to their originally supply points after completing their evacuation task. However, only the first stage can be directly treated as the traditional transportation problem, which cannot be applied for the second stage that feeder-buses operate circularly between the demand points and the third stage that feeder-buses return back.

**Fig 6:**
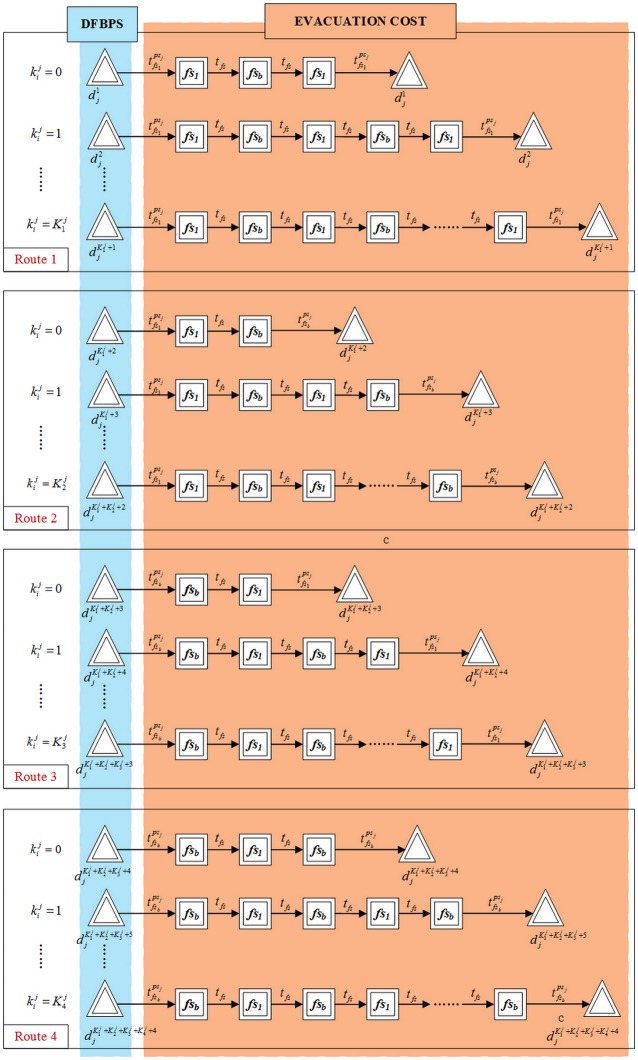
Transformation process of dummy feeder-bus system.

In order to transfer feeder-bus dispatch planning model under the URT corridor’s emergency into a traditional transportation problem, a dummy feeder-bus dispatch system is proposed theoretically, as is shown in Fig 6. The dummy feeder-bus dispatch system includes the sets of dummy feeder-bus parking spots, dummy feeder-bus stations and dummy feeder-buses. It is assumed that in the dummy feeder-bus dispatch system, the whole operation route of dummy feeder-buses is from the dummy feeder-bus parking spots to the dummy feeder-bus stations. Therefore, the feeder-bus dispatch problem under the URT corridor’s emergency can be simply re-described as follows: all the dummy feeder-buses are dispatched from dummy feeder-bus parking spots to dummy feeder-bus stations.

### Basic features of the Dummy feeder-bus system

(1) Analysis of the dummy feeder-bus parking spots

Within the time window, the number of dummy feeder-bus parking spots for each subsistent feeder-bus parking spot is synchronously determined by the number of operation routes and the maximum operation times of dispatched feeder-buses, which should be set as 4 and $1\text{+}K_{r}^{ps}$, respectively. In particular, as is shown in Fig 6, the number of dummy feeder-bus parking spots of the subsistent feeder-bus parking spot, *ps*_*j*_, is $1+K_{r}^{ps_{j}}$ for each operation route. To simplify the expression, let *j* = *ps*_*j*_, which can both represent the *j* subsistent feeder-bus parking spot. Therefore, Let $D_{j}=\left\{D_{j}|d_{j}=d_{j}^{1}\;,..,\;d_{j}^{K_{1}^{j}+K_{2}^{j}+K_{3}^{j}+K_{4}^{j}+4}\right\}$ represent the sets of dummy feeder-bus parking spots of the subsistent feeder-bus parking spot, *j*, where *j* ∈ *PS*, 1≤*j*≤*a*. The maximum number of dummy feeder-buses which can be dispatched from each dummy feeder-bus parking spot is the equal to that of its subsistent feeder-bus parking spot. However, due to the capacity of subsistent feeder-buses, there will be an additional constrain. As is shown in Eq (21), for each subsistent feeder-bus parking spot, *j* ∈ *PS*, 1≤*j*≤*a*, the total number of dispatched feeder-buses from its dummy feeder-bus parking spots, $\sum_{1\leq i\leq K_{1}^{j}+K_{2}^{j}+K_{3}^{j}+K_{4}^{j}+4}x_{j}^{i}$, should be smaller than its real capacity, $C^{ps_{j}}$.

$$\sum_{1\leq i\leq K_{1}^{j}+K_{2}^{j}+K_{3}^{j}+K_{4}^{j}+4}x_{j}^{i}\leq C^{ps_{j}}$$ (21)

(2) Analysis of dummy feeder-buses

The dummy feeder-buses and their subsistent dispatched feeder-buses have a strong correspondence relationship. The distribution of dispatched dummy feeder-buses is determined by the operation routes and the circular operation times of their subsistent feeder-buses. Particularly, when a subsistent feeder-bus operate circularly for *w* times along Route 1, it is assumed that a dummy feeder-bus will be dispatched from dummy parking spot $d_{j}^{\text{w+}1}$, where $0\leq w\leq K_{1}^{j}$; when operating circularly for *w* times along Route 2, it is assumed that a dummy feeder-bus will be dispatched from dummy parking spot $d_{j}^{w+K_{1}^{j}\text{+2}}$, where $0\leq w\leq K_{2}^{j}$; when operating circularly for *w* times along Route 3, it is assumed that a dummy feeder-bus will be dispatched from dummy parking spot $d_{j}^{w+K_{1}^{j}\text{+}K_{2}^{j}+3}$, where $0\leq w\leq K_{3}^{j}$; and when operating circularly for *w* times along Route 4, it is assumed that a dummy feeder-bus will be dispatched from dummy parking spot $d_{j}^{w+K_{1}^{j}\text{+}K_{2}^{j}+K_{3}^{j}\text{+4}}$, where $0\leq w\leq K_{\text{4}}^{j}$. The design seating capacity of dummy feeder-buses is set as that of their subsistent feeder-buses, as is shown in Eq (22).

$$C_{b}^{*}{=\text{C}}_{b}$$ (22)

(3) Analysis of travel cost

Each dummy feeder-bus dispatched from dummy feeder-bus parking spot $d_{j}^{i}$ incurs a variable cost of $c_{j}^{i}$. Obviously, the dummy feeder-buses dispatched from different dummy feeder-bus parking spots will lead to different costs. In assumption 2, the time feeder-buses running from feeder-bus station *fs* to *fs*′ is equal to that from *fs*′ to *fs*. Therefore, from Fig 6, it can be found that when a dummy feeder-bus is dispatched from dummy feeder-bus parking spot $d_{j}^{i}$, where $1\leq i\leq K_{\text{1}}^{j}\text{+1}$, the cost is equal to $2*i*T+2*t_{fs_{1}}^{j}$; when $K_{\text{1}}^{j}+2\leq i\leq K_{\text{1}}^{j}\text{+}K_{2}^{j}\text{+2}$, the cost is $t_{fs_{1}}^{j}+[2*(i-K_{\text{1}}^{j}-1)-1]*T+t_{fs_{b}}^{j}$; when $K_{\text{1}}^{j}\text{+}K_{2}^{j}\text{+3}\leq i\leq K_{\text{1}}^{j}\text{+}K_{2}^{j}\text{+}K_{3}^{j}\text{+3}$, the cost is $t_{fs_{b}}^{j}+[2*(i-K_{i}^{j}-K_{2}^{j}\text{-2)}-1]*T+t_{fs_{1}}^{j}$; when $K_{\text{1}}^{j}\text{+}K_{2}^{j}\text{+}K_{3}^{j}\text{+4}\leq i\leq K_{\text{1}}^{j}\text{+}K_{2}^{j}\text{+}K_{3}^{j}\text{+}K_{4}^{j}\text{+4}$, the cost is $2*(i-K_{\text{1}}^{j}-K_{2}^{j}-K_{3}^{j}\text{-3)}*T+2*t_{fs_{b}}^{j}$, as listed in Eq (23).

$$c_{j}^{i}=\left\{ \begin{array}{ll} {}[2*i*T+2*t_{fs_{1}}^{j}]*x_{j}^{i}, & 1\leq i\leq K_{\text{1}}^{j}\text{+1}\\ \{t_{fs_{1}}^{j}+[2*(i-K_{\text{1}}^{j}\text{-1)}-1]*T+t_{fs_{b}}^{j}\}*x_{j}^{i}, & K_{\text{1}}^{j}+2\leq i\leq K_{\text{1}}^{j}\text{+}K_{2}^{j}\text{+2}\\ \{t_{fs_{b}}^{j}+[2*(i-K_{i}^{j}-K_{2}^{j}\text{-2)}-1]*T+t_{fs_{1}}^{j}\}*x_{j}^{i}, & K_{\text{1}}^{j}\text{+}K_{2}^{j}\text{+3}\leq i\leq K_{\text{1}}^{j}\text{+}K_{2}^{j}\text{+}K_{3}^{j}\text{+3}\\ {}[2*(i-K_{\text{1}}^{j}-K_{2}^{j}-K_{3}^{j}\text{-3)}*T+2*t_{fs_{b}}^{j}]*x_{j}^{i}, & K_{\text{1}}^{j}\text{+}K_{2}^{j}\text{+}K_{3}^{j}\text{+4}\leq i\leq K_{\text{1}}^{j}\text{+}K_{2}^{j}\text{+}K_{3}^{j}\text{+}K_{4}^{j}\text{+4} \end{array}\right.$$ (23)

(4) Analysis of supporting evacuation capacity

Dummy feeder-buses dispatched from different dummy feeder-bus parking spot will provide different supporting evacuation capacity for both directions. As is shown in Fig 6, when $1\leq i\leq K_{\text{1}}^{j}\text{+1}$, each dummy feeder-bus can evacuate i*C_b_*φ_*b*_ passengers in both the up direction and down direction; when $K_{\text{1}}^{j}+2\leq i\leq K_{\text{1}}^{j}\text{+}K_{2}^{j}\text{+2}$, the evacuation capacity of each dispatched dummy feeder-bus is $(i-K_{1}^{j}\text{-1)}*C_{b}*\varphi_{b}$ for the up direction and $(i-K_{\text{1}}^{j}\text{-2)}*C_{b}*\varphi_{b}$ for the down direction, respectively; when $K_{\text{1}}^{j}\text{+}K_{2}^{j}\text{+3}\leq i\leq K_{\text{1}}^{j}\text{+}K_{2}^{j}\text{+}K_{3}^{j}\text{+3}$, the capacity is $(i-K_{\text{1}}^{j}-K_{2}^{j}\text{-3)}*C_{b}*\varphi_{b}$ for the up direction and $(i-K_{\text{1}}^{j}-K_{2}^{j}\text{-2)}*C_{b}*\varphi_{b}$ for the down direction, respectively; when $K_{\text{1}}^{j}\text{+}K_{2}^{j}\text{+}K_{3}^{j}\text{+4}\leq i\leq K_{\text{1}}^{j}\text{+}K_{2}^{j}\text{+}K_{3}^{j}\text{+}K_{4}^{j}\text{+4}$, the capacity is $(i-K_{\text{1}}^{j}-K_{2}^{j}-K_{3}^{j}\text{-3)}*C_{b}*\varphi_{b}$ for the up direction and $(i-K_{\text{1}}^{j}-K_{2}^{j}-K_{3}^{j}-3\text{)}*C_{b}*\varphi_{b}$ for the down direction, respectively. Therefore, the evacuation capacity of each dispatched dummy feeder-bus from dummy parking feeder-bus parking spot, $d_{j}^{i}$ can be listed in Table 4.

**Table 4:** The Supporting Evacuation Capacity of Each Dummy Feeder-Bus.

The serial number of dummy feeder-bus parking spots	Up direction	Down direction
$1\leq i\leq K_{\text{1}}^{j}\text{+1}$	i*C_b_*φ_*b*_	i*C_b_*φ_*b*_
$K_{\text{1}}^{j}+2\leq i\leq K_{\text{1}}^{j}\text{+}K_{2}^{j}\text{+2}$	$(i-K_{1}^{j}\text{-1)}*C_{b}*\varphi_{b}$	$(i-K_{\text{1}}^{j}\text{-2)}*C_{b}*\varphi_{b}$
$K_{\text{1}}^{j}\text{+}K_{2}^{j}\text{+3}\leq i\leq K_{\text{1}}^{j}\text{+}K_{2}^{j}\text{+}K_{3}^{j}\text{+3}$	$(i-K_{\text{1}}^{j}-K_{2}^{j}\text{-3)}*C_{b}*\varphi_{b}$	$(i-K_{\text{1}}^{j}-K_{2}^{j}\text{-2)}*C_{b}*\varphi_{b}$
$K_{\text{1}}^{j}\text{+}K_{2}^{j}\text{+}K_{3}^{j}\text{+4}\leq i\leq K_{\text{1}}^{j}\text{+}K_{2}^{j}\text{+}K_{3}^{j}\text{+}K_{4}^{j}\text{+4}$	$(i-K_{\text{1}}^{j}-K_{2}^{j}-K_{3}^{j}\text{-3)}*C_{b}*\varphi_{b}$	$(i-K_{\text{1}}^{j}-K_{2}^{j}-K_{3}^{j}-3\text{)}*C_{b}*\varphi_{b}$

### Modeling based on the concept of dummy parking spot

Based on the concept of dummy parking spot and above analyses, the original model can be transformed into the following ILP model.

**Objective function based on the concept of dummy parking spot**:

The objective of the dispatched scheme is to minimize the total cost, i.e., the total service time of all the dispatched feeder-buses, as shown in Eq (24).

$$Min\;C=\;\sum_{1\leq j\leq a,\;j\in PS}\sum_{1\leq i\leq K_{\text{1}}^{j}\text{+}K_{\text{2}}^{j}\text{+}K_{\text{3}}^{j}\text{+}K_{\text{4}}^{j}\text{+4}}c_{j}^{i}$$ (24)

**Constrains based on the concept of dummy parking spot**:

(1) Limitation of dummy feeder-bus parking spot capacity

Constraint (25) describes that the total number of dummy feeder-buses dispatched from all the dummy feeder-bus parking spots of a subsistent feeder-bus parking spot must be less than the useable number of feeder-buses in the subsistent feeder-bus parking spot.

$$\sum_{1\leq i\leq K_{\text{1}}^{j}\text{+}K_{2}^{j}\text{+}K_{3}^{j}\text{+}K_{4}^{j}\text{+4}}x_{j}^{i}\leq C^{j}\;where\;1\leq j\leq a$$ (25)

(2) Capacity of dummy dispatched feeder-bus constraint

Constraints (26) and (27) restrain that the supporting capacity of all the dispatched dummy feeder-buses should be more than the feeder-bus demand of each direction.

$$C_{b}*\varphi_{b}*\sum_{1\leq j\leq a,\;j\in PS}\left(\begin{array}{l} \sum_{1\leq i\leq K_{\text{1}}^{j}\text{+1}}i*x_{j}^{i}+\\ \sum_{K_{\text{1}}^{j}+2\leq i\leq K_{\text{1}}^{j}\text{+}K_{2}^{j}\text{+2}}(i-K_{\text{1}}^{j}-1)*x_{j}^{i}+\\ \sum_{K_{\text{1}}^{j}\text{+}K_{2}^{j}\text{+3}\leq i\leq K_{\text{1}}^{j}\text{+}K_{2}^{j}+K_{3}^{j}\text{+3}}(i-K_{\text{1}}^{j}-K_{2}^{j}-3)*x_{j}^{i}+\\ \sum_{K_{\text{1}}^{j}\text{+}K_{2}^{j}\text{+}K_{3}^{j}\text{+4}\leq i\leq K_{\text{1}}^{j}\text{+}K_{2}^{j}\text{+}K_{3}^{j}\text{+}K_{4}^{j}\text{+4}}(i-K_{\text{1}}^{j}-K_{2}^{j}-K_{3}^{j}-3)*x_{j}^{i} \end{array}\right)\geq q_{\text{max}}^{1}$$ (26)

$$C_{b}*\varphi_{b}*\sum_{1\leq j\leq a,\;j\in PS}\left(\begin{array}{l} \sum_{1\leq i\leq K_{\text{1}}^{j}\text{+1}}i*x_{j}^{i}+\\ \sum_{K_{1}^{j}+2\leq i\leq K_{\text{1}}^{j}\text{+}K_{2}^{j}\text{+2}}(i-K_{\text{1}}^{j}-2)*x_{j}^{i}+\\ \sum_{K_{\text{1}}^{j}\text{+}K_{2}^{j}\text{+3}\leq i\leq K_{\text{1}}^{j}\text{+}K_{2}^{j}\text{+}K_{3}^{j}\text{+3}}(i-K_{\text{1}}^{j}-K_{2}^{j}-2)*x_{j}^{i}+\\ \sum_{K_{\text{1}}^{j}\text{+}K_{2}^{j}\text{+}K_{3}^{j}\text{+4}\leq i\leq K_{\text{1}}^{j}\text{+}K_{2}^{j}\text{+}K_{3}^{j}\text{+}K_{4}^{j}\text{+4}}(i-K_{\text{1}}^{j}-K_{2}^{j}-K_{3}^{j}-3)*x_{j}^{i} \end{array}\right)\geq q_{\text{max}}^{2}$$ (27)

(3) Nonnegative integer constraints of the number of feeder-buses

Constraint (28) means that the number of feeder-buses dispatched from dummy parking spot $d_{j}^{i}$ is nonnegative integer.

$$x_{j}^{i}\in N$$ (28)

## Case Study

To evaluate the proposed model and solution approach, we used the Nanjing URT as a case study, the operating mileage of which was 225.4 km until January, 2016, including 6 lines and 121 stations. An emergency of infrastructure malfunctions occurred between Xiaoweilin Station and Xiamafang Station along Nanjing URT Line #2 at 2:40 pm, on August 22^th^, 2011. There were totally 26 stations along URT Line #2. If canceling the whole line operation under the emergency, it would bring substantial inconvenience to passengers and lead to huge economic losses. Since only several nearby stations were affected by the emergency, the method proposed in this paper is quite suitable to solve such an emergency response issue.

### Main features of the case study

**Fig 7:**
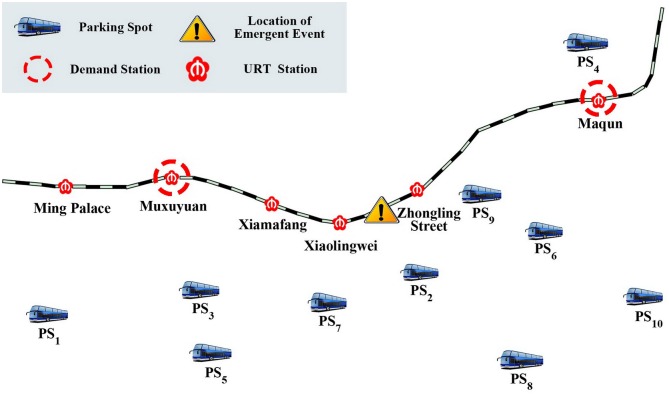
Nanjing URT Line #2 and surrounding parking spots.

Nanjing URT Line #2 connects Nanjing’s central business district and the suburb with a high commuting demand. To relieve the emergency influence, the contingency plan of feeder-bus should be launched. Youqiaofang Station and Jingtianlu Station are the two endpoint stations. The direction from Youqiaofang Station to Jingtianlu Station is defined as up direction, and inversely down direction. As shown in Fig 7, Muxuyuan Station and Maqun Station were equipped with the turn-back line, which are defined as demand stations where feeder-buses are dispatched to. Xiamafang Station, Zhongling Stree Station and Xiaolingwei Station are defined as middle stations. *PS* = {*ps* | *ps* = 1, 2, 3, …, 10} represent a finite set of feeder-bus parking spots.

In this study, the time window is targeting as two hours and the journey time between two adjacent stations is fixed, five minutes. All the basic information about URT system, the feeder-bus parking spots and road system was listed as follows:

**The parameters related with the URT system are set as follows**:

Train departure interval of the small routings: *h* = 5 min;

Capacity of each URT train: *C*_*u*_ = 2460;

Load factor of URT train: *φ*_*u*_ = 0.7;

The number of original stranded passengers in each URT station *s* when the emergent event occurs: *Q*_*s*_ = 100;

The number of passengers arriving at station *s* from outside per hour: *q* = 300

**The parameters related to the feeder-bus are set as follows**:

Design seating capacity: *C*_*b*_ = 80;

Load factor of feeder-bus: *φ*_*b*_ = 1.2;

Minimum journey time between feeder-bus stations *fs*_1_ and *fs*_*b*_: *t*_*fs*_ = 25min;

**The parameters related to the feeder-bus parking spot are set as follows**:

The total number, *C*^*ps*^, of useable feeder-buses in the feeder-bus parking spot, *ps*, along with the minimum journey time, $t_{fs}^{ps}$, from parking spot *ps* to feeder-bus station *fs* are listed in the Table 5.

**Table 5:** Information about the Buses in the Parking Spots.

Parking Spot *ps*	$t_{fs_{\text{1}}}^{ps}$ (min)	$t_{fs_{\text{2}}}^{ps}$ (min)	*C*^*ps*^ (buses)
1	8	32	7
2	9	9	7
3	5	29	7
4	26	4	7
5	12	33	7
6	20	6	7
7	11	18	7
8	25	23	7
9	13	7	7
10	30	13	7

According to the Eqs (1) to (10), in the up direction, the evacuation demand of feeder-bus station, Muxuyuan Station, is $q_{fs_{1}}^{1}=\text{6755}$. The evacuation demands of feeder-bus stations, Xiamafang Station, Xiaolinwei Station and Zhonglingjie Station, are $q_{fs_{2}}^{1}=\text{5742}$, $q_{fs_{3}}^{1}=\text{5222}$ and $q_{fs_{4}}^{1}=\text{4845}$, respectively. Similarly, in the down direction, the evacuation demand of feeder-bus station, Maqun Station, is $q_{fs_{5}}^{2}=\text{6969}$. The evacuation demands of feeder-bus stations, Zhonglingjie Station, Xiaolinwei Station and Xiamafang Station, are $q_{fs_{4}}^{2}=\text{7540}$, $q_{fs_{3}}^{2}=\text{8286}$ and $q_{fs_{2}}^{2}=\text{9348}$, respectively. Therefore, the maximum section passenger volume of the up direction $q_{\text{max}}^{1}$ is 6755 and that of the down direction is $q_{\text{max}}^{2}$ 9348.

The proposed feeder-bus dispatch optimization model is coded in C# and solved by LINGO 11, and the computational experiments were run on a 2.9 GHz Core i5 PC with 4 GB of RAM. The proposed model can be solved within a few seconds.

### Computational results and comparison with the Genetic Algorithm

Table 6 lists the optimal computational results based on the above information. The results clearly show the optimal feeder-bus dispatched scheme, i.e., feeder-buses in each parking spot should operate along with alternative route and operate for a certain number of times. As previously mentioned, each feeder-bus has four alternative routes which are represented by (*,*) in Table 6. The codes in parentheses represent the two demand stations, the former being the departure station and the other being the return station. The number of dispatched feeder-buses along with the operation times was also listed.

**Table 6:** Computational Results of the Feeder-Bus Dispatch Scheme.

Parking spot	*t* = 2				*t* = 2.5				*t* = 3				*t* = 3.5			
	Four alternative routes															
	(1, 1)	(1, 2)	(2, 1)	(2, 2)	(1, 1)	(1, 2)	(2, 1)	(2, 2)	(1, 1)	(1, 2)	(2, 1)	(2, 2)	(1, 1)	(1, 2)	(2, 1)	(2, 2)
1	7(1)															
2			7(1)				7(2)				7(2)				7(3)	
3	7(1)				7(1)											
4				7(1)				7(1)				1(1), 5(2)				
5																
6			6(1)	1(1)			6(1)	1(1)			7(2)				7(3)	
7			7(1)				7(1)				6(2)				2(2), 2(3)	
8																
9			7(1)				7(2)				7(2)				7(3)	
10																

**Note:** In the calculation results, the numbers not in parentheses were the number of dispatched feeder-buses, and the numbers in parentheses were their circulation operation times.

Given that the time window is two hours, the best dispatched scheme can be described as follows: the Parking Spots 1 and 3 respectively dispatch seven feeder-buses to Muxuyuan Station and the feeder-buses also return back from Muxuyuan Station; the Parking Spots 2, 6, 7 and 9 respectively dispatch seven, six, seven and seven feeder-buses to Maqun Station and the feeder-buses return back from Muxuyuan Station; the Parking Spot 4 and 6 respectively dispatch seven and one feeder-buses to Maqun Station and the feeder-buses also return back from Maqun Station.

As previously mentioned, many researchers have applied the heuristic algorithm to solve the UFSIP. In order to highlight the performance of this paper in improving the feeder-bus utilization efficiency, the Genetic Algorithm (GA) was applied to carried out same experiments in the same computer. What should be noted is that, due to the instability of the solution quality and convergence speed of GA, 20 experiments were carried out, and the best one was chosen to be compared with the results obtained by our approach. Detail comparison results are as listed in Table 7. The results obviously show that the solution approach in this study has the advantages over GA in improving the solution quality and shortening the computational time. Particularly, compared with the optimal scheme obtained by GA, 19 feeder-buses were saved and total evacuation time of all the feeder-buses were 1755 minutes smaller. Moreover, the computation time of our approach is only 12 milliseconds, which is much smaller than that of GA.

**Table 7:** Results of the Comparison between Our Approach and GA.

	Our approach	GA	Difference
The number of dispatched feeder-buses	49	68	19
The total evacuation time (minutes)	5100	6855	1755
The computational time (millisecond)	12	6056	6044

### Assessing impact of the time window

To assess the impact of time window on the feeder-bus dispatch scheme, we assigned a fixed capacity of seven feeder-buses in each parking spot, a fixed evacuation demand (up direction: 6755 passengers, down direction: 9348 passengers) and the journey time between two adjacent middle stations as five minutes. In this study, we increased the time window, *t*, from 0.5 hour to 3.5 hours. When the time window is less than 2 hours, there is no feasible solution, indicating that the evacuation task is impossibly completed within the one-hour evacuation time window.

**Fig 8:**
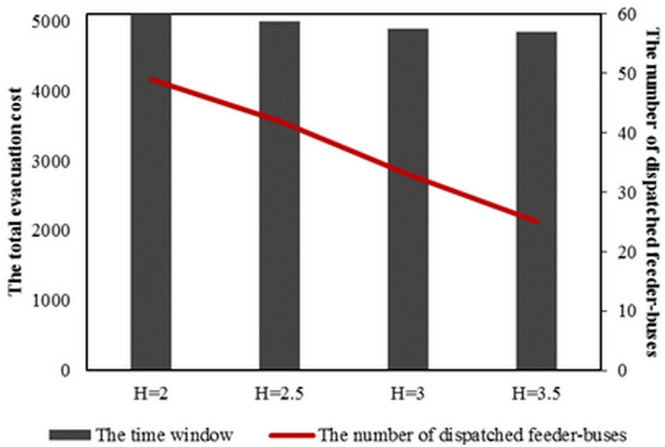
Total evacuation cost and number of dispatched feeder-buses with different time windows.

For the feasible solutions, the total evacuation cost along with the number of dispatched feeder-buses is shown in Fig 8. The results reveal that with the increase of time window, the total evacuation cost decreases slightly and the number of dispatched feeder-buses decreases sharply. The reason can be described as follows. The operating time of feeder-buses can be classified into two types, namely: the time that feeder-buses use to depart from and return back to parking spots; and the time that feeder-buses use to operate between two demand stations. The former one is fixed which will not change with the time window. Inversely, the latter will be larger with the increase of time window. Moreover, passengers can only be evacuated during the latter time period. Therefore, as listed in Table 6, when the time window becomes longer, the dispatched feeder-buses will be more likely to operate for more operation times along the feeder-bus line. As a result, to satisfy the same evacuation demand, less feeder-buses will be needed with the increase of required time window.

### Determining the feeder-bus parking spot capacity

**Fig 9:**
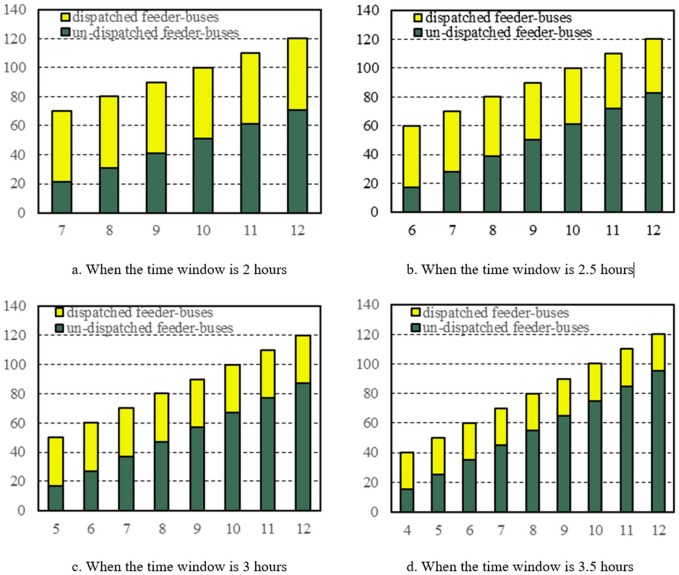
Number of dispatched and un-dispatched feeder-buses with different capacity of feeder-bus parking spots.

Sensitivity analysis of feeder-bus parking spot capacity is conducted to help the emergency response decision makers find a reasonable feeder-bus fleet size. Based on above findings, the variable time window has a strong impact on the feeder-bus dispatch scheme. Therefore, the optimal feeder-bus parking spot capacities were explored for different time windows. The results reveal that, when the time window is short and the parking spot capacity is small, the model will have no feasible solution. It is because limited by the short time window, even all the feeder-buses are dispatched, the supporting capacity is still not enough. From Fig 9(a) to 9(d), it can be found that in order to ensure the model solvable when the time window is 2 hours, the least capacity of each parking spot is 7, and then when the time window increases by 0.5 hour gradually, each parking spot’s least capacity can decrease by one bus.

Fig 9 illustrates relationship between the numbers of un-dispatched and dispatched feeder-buses under different time windows and parking spot capacities. An interesting finding is that the number of dispatched feeder-buses does not show an obvious change with the increase of parking spot capacity and time window. It means that simply increasing the parking spot capacity will cause huge waste for the emergent bus utilization. For the evacuation demand in this paper, the optimal capacity of parking spot should be set from four to seven based on different time windows.

### Investigating the effectiveness of evacuation demand

**Fig 10:**
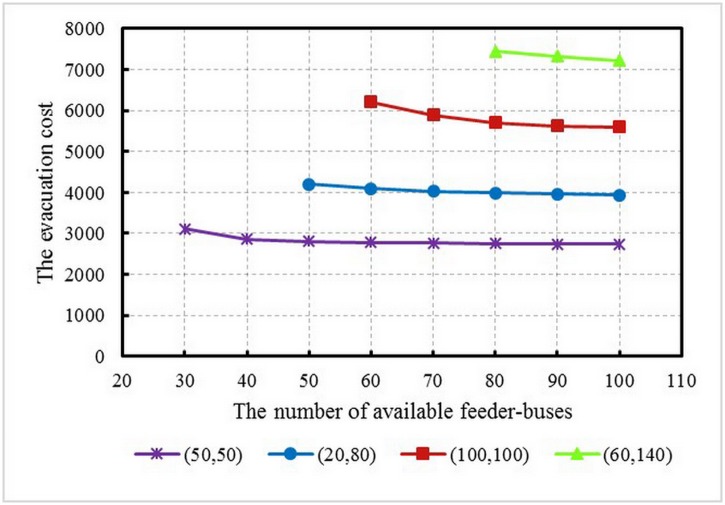
Total evacuation cost with different evacuation demand.

In the URT system, passenger volumes fluctuate strongly during different time periods of a day, and always appear the characteristics of tidal phenomenon. To investigate the impact of disruptions happening during different time periods as well as the non-uniformity of evacuation demand on the feeder-bus dispatch scheme, the computational experiments under four circumstances were conducted to compare the total evacuation cost and the number of dispatched feeder-buses: (1) the evacuation demand of two demand stations are both 50 feeder-buses, i.e., non-peak period vs. uniformity; (2) the evacuation demand of two demand stations are respectively 20 and 80 feeder-buses, i.e., non-peak period vs. non-uniformity; (3) the evacuation demand of two demand stations are both 100 feeder-buses, i.e., peak period vs. uniformity; and (4) the evacuation demand of two demand stations are respectively 60 and 140 feeder-buses, i.e., peak period vs. non-uniformity. It should be noted that due to the complexity of calculation, the demand of feeder-buses directly was given instead of the passenger evacuation demand. The number of middle stations is set as 5, and the time window is set as 2 hours. Fig 10 shows the total evacuation cost for the tested four types of evacuation demand. It can be seen that the evacuation demand significantly influenced the total evacuation cost during both of peak and non-peak periods: when the total evacuation demand of two demand stations is 100, the total evacuation cost is about 3000; when the total evacuation demand increase to 200, the total evacuation cost will be more than 5000. By comparing between the demands of (50, 50) and (20, 80), the total evacuation cost of the latter is larger than that of the former. Additionally, the stability of solution is better when the demands are both 50. Similar conclusions can also be found when comparing between the demands of (100, 100) and (60, 140). The results reveal that the evacuation demand uniformity of the two demand stations significantly influenced the optimal solution. When the evacuation demand difference between two stations increased, the more feeder-buses will be needed. The results could help decision makers better prepare response actions when the URT disruption happens over different time periods.

## Conclusion

The feeder-bus dispatch problem under URT emergency profoundly affects public security and safe operation of the public transportation. When an emergent event occurs at a point along a certain URT line and breaks its operation, the nearby feeder-buses can be dispatched to the influenced stations and served as a ferry system to connect the disrupted URT line. To realize a trains-buses-trains cooperation mode during emergency, in this study, a feeder-bus dispatch planning model is proposed to minimize the total travelling time of feeder-buses when targeting a stranded passenger evacuation time period. In the process of model solution, a concept of dummy feeder-bus system is proposed to translate the non-linear integer programming (NLIP) into the traditional linear integer programming (LIP). For realistic applications, some other specific factors can be calibrated and integrated into the model, e.g., modeling the journey time between two demand stations in more detail, considering the road condition, running speed, the duration of passengers getting on and off in stations, etc. Using the model, the most sensitive time window and the capacity of feeder-bus parking spot according to different evacuation demands in URT corridors can be identified, and the transportation emergency management and resource allocation can be more effectively implemented by operation departments.

In order to improve the practical applicability of the proposed model, some further studies in two areas are suggested. Firstly, we only focused on modeling the optimal feeder-bus dispatch scheme for an URT corridor in this study. However, the model is not suitable for emergency occurring at transfer stations in an URT network. Introducing transfer stations into the model can cause the scale of variables to grow substantially and lead to a challenge for developing the optimal solution of algorithms. Therefore, more complex URT networks are suggested for the further model exploration in future research. Secondly, the objective of proposed model is to minimize the total travel time of feeder-buses, which represents the level of service provided by the stakeholder of bus companies. Actually, there are typically four sets of stakeholders associated with the decision-making problem of feeder-bus dispatch, including emergency response planners, bus service providers, evacuation users, and the other nonusers [26]. However, the costs of the four sets of stakeholders are incompatible in some cases so that it is difficult to combine the individual evacuation user costs and bus operator costs in a single objective function. Moreover, this study mainly focuses on feeder-bus dispatch scheme planning rather than the feeder-bus operation timetabling, and thus, the detailed individual travel time of each service user cannot be obtained by the proposed model. Therefore, it is recommended to develop a multi-objective model for the feeder-buses operational timetable design in a future study which can incorporate the costs of both bus operators and evacuation users.
